# Parasomnias: A Comprehensive Review

**DOI:** 10.7759/cureus.3807

**Published:** 2018-12-31

**Authors:** Shantanu Singh, Harleen Kaur, Shivank Singh, Imran Khawaja

**Affiliations:** 1 Pulmonary Medicine, Marshall University School of Medicine, Huntington, USA; 2 Neurology, Univeristy of Missouri, Columbia, USA; 3 Internal Medicine, Maoming People's Hospital, Maoming, CHN

**Keywords:** parasomnia, sleep walking, confusional arousals, sleep terror, nightmares, rem behavior disorder, sleep paralysis, rem parasomnias, nrem parasomnias

## Abstract

Parasomnias are a group of sleep disorders characterized by abnormal, unpleasant motor verbal or behavioral events that occur during sleep or wake to sleep transitions. Parasomnias can occur during non-rapid eye movement (NREM) and rapid eye movement (REM) stages of sleep and are more commonly seen in children than the adult population. Parasomnias can be distressful for the patient and their bed partners and most of the time, these complaints are brought up by their bed partners because of the possible disruption in their quality of sleep. As clinicians, it is crucial to understand the characteristics of various parasomnias and address them with detailed sleep history and essential diagnostic approach for proper evaluation.

The review aims to highlight the epidemiology, pathophysiology and clinical features of various types of parasomnias along with the appropriate diagnostic and pharmacological approach.

## Introduction and background

Parasomnias are a group of sleep disorders that are characterized by abnormal, unpleasant motor, verbal or behavioral events that occur during sleep or wake to sleep transitions [1]. The term ‘parasomnia’ was first coined by a French researcher Henri Roger in 1932 [2]. This nomenclature is originally derived from the Greek word ‘para’ meaning beside or along the side of and Latin term ‘somnus’ meaning sleep. Parasomnias are more common in children than in the adult population. They can be seen in both non-rapid eye movement (NREM) and rapid eye movement (REM) sleep states and are classified separately by the Diagnostic and Statistical Manual of Mental Disorders-5 (DSM-5) and International Classification of Sleep Disorders-3 (ICSD-3). Parasomnias may occur alone or in the setting of trauma, psychiatric illness, other sleep-related disorders, Parkinson’s disease and spinocerebellar ataxia. The REM and NREM parasomnias may occur together and are termed as parasomnia overlap syndrome. A complete sleep history and diagnostic workup including polysomnography is important to rule out the differentials and reach a conclusive diagnosis of parasomnia.

The review aims to highlight the epidemiology, pathophysiology and clinical features of various types of parasomnias, along with an appropriate diagnostic workup and pharmacological approach.

## Review

Epidemiology

Parasomnias are more often seen in children than in the adult population. In children, the NREM parasomnias are more common than REM parasomnia. Amongst these, the prevalence rate of confusional arousals in children of age group 3 to 13 years is 17.3% and more than 15 years is 6.9% [3]. The prevalence rate of sleepwalking under 12 years of age is 17% and that of sleep terror is 6.5% [4]. Nightmares are seen in 10% to 50% of the pediatric population. Parasomnias are more frequently seen in children with underlying neurologic and psychiatric issues like epilepsy, attention-deficit hyperactive disorder (ADHD) or developmental issues [4-6].

In adults, the lifetime prevalence of various parasomnia ranges from 4% to 67% and is further summarized in Table 1.

**Table 1:** Prevalence of parasomnia in pediatric and adult population NREM: non-rapid eye movement, REM: rapid eye movement

Parasomnia	Adults lifetime prevalence in %	Children prevalence in % (under 15 years of age)
NREM		
Sleepwalking	22.4	17.0
Confusional arousal	18.5	17.3
Sleep terror	10.4	6.5
Sleep-related eating disorder	4.5	
Sexual act during sleep/sexsomnia	7.1	
REM		
REM behavior disorder	15.0	
Sleep-related groaning/Catathrenia	31.3	
Nightmare	66.2	10-50

No gender difference has been reported in sleepwalking, sleep terrors or confusional arousals [7]. However, nightmares are reported to be more common in the female population [8]. Sexsomnia is seen more often in the male population. Likewise, REM behavior disorders (RBD) are reported to be more in male patients over the age of 50 years. The RBDs are also likely to be linked to neurodegenerative disorders like alpha(α)-synucleinopathies (Parkinson’s disease, Lewy body dementia and multiple system atrophy) [9]. Besides, a significantly higher prevalence rate of parasomnia has been reported in psychiatric conditions, with nightmare being 38.9%, sleep paralysis 22.3%, sleep-related eating disorders 9.9%, sleepwalking 8.5% and RBD 3.8% [10].

Pathophysiology

The various stages of normal sleep cycle include the transition from wakefulness to NREM sleep and REM sleep. The NREM sleep occurs in the first half of the night and typically includes stage one (transitional stage), stage two, stage three and stage four. The disorders of arousal are presumed to occur because of the incomplete transition or sleep-wake boundary dyscontrol between wakefulness and stages of sleep. The NREM parasomnias are most often seen during the slow-wave sleep (stage three) but can arise in stage two sleep as well [11]. Most of the pediatric parasomnia are benign disorders and occur because of the immaturity of sleep-wake boundary regulations [12].

Likewise, the REM parasomnias are considered to occur because of the admixture of wakefulness and REM sleep. The possible explanation for the increased motor activity in RBD is because of the deafferentation of the locomotor centers (at the spinal and supraspinal levels) that result in oroalimentary automatism, bruxism and ambulatory behavior [13].

The disorders of arousal are also presumed to be triggered by sleep deprivation, sedating medications, sleep fragmentation (pain, restless leg syndrome, periodic limb movement and obstructive sleep apnea), febrile illness and alcohol [1]. The parasomnia overlap syndrome can be seen secondary to narcolepsy, rhombencephalitis, multiple sclerosis, brain tumors, spinocerebellar ataxia type three (Machado-joseph syndrome), psychiatric disorders, substance abuse and alcohol withdrawal [14-15]. NREM and REM parasomnias are also noted in Anti-IgLON5 disease. This neurological disease was recently discovered in 2014 and includes a cascade of neurodegenerative, neuroimmunological, sleep and movement disorder aspects. Sleep disorders noted in anti-IgLON5 disease are REM parasomnia, NREM parasomnia, obstructive sleep apnea and stridor. The disease has a higher association with human leucocyte antigen (HLA)-DRB1*10:01 and HLA-DQB1*05:01 and results from antibodies against IgLON5 (neuronal cell adhesion protein) [16].

Besides, the genetic factors linking to parasomnia include a higher prevalence of HLA B1*05:01 and HLA DQB1*04 in NREM parasomnia [17]. Another genetic component identified is an autosomal dominant trait for sleepwalking on chromosome 20 [18].

Classification of parasomnias

The parasomnias are classified according to ICSD-3 into the following categories, as shown in Table 2 [19]:

**Table 2:** Classification of parasomnias according to International Classification of Sleep Disorders-3 NREM: non-rapid eye movement, REM: rapid eye movement

A	NREM-related parasomnia
1.	Confusional arousals
2.	Sleepwalking
3.	Sleep terrors
4.	Sleep-related eating disorder
B	REM-related parasomnia
1.	REM sleep behavior disorder
2.	Recurrent isolated sleep paralysis
3.	Nightmare disorder
C	Other parasomnias
1.	Exploding head syndrome
2.	Sleep-related hallucinations
3.	Sleep enuresis
4.	Parasomnia due to a medical disorder
5.	Parasomnia due to medical or substance abuse
6.	Parasomnia, unspecified

Clinical features of different types of parasomnia

Confusional Arousals

Confusional arousals are partial awakenings during the slow-wave sleep (stage three) and are also termed as sleep drunkenness, Elpenor's syndrome or morning sleep inertia [7]. In this, the individual is confused, disoriented to time and space and may involve some automatic behavior like opening eyes, mumbling, without any motor activity or sympathetic hyperactivity lasting for a few minutes to hours. The individual has total amnesia of the event. Confusional arousals can be triggered by a sedative-hypnotic use or alcohol abuse. Confusional arousals are usually benign and more common in children than in the adult population [11].

Sleep Walking

Sleepwalking or somnambulism is an arousal disorder resulting in ambulatory behavior in stage three of sleep. During these nocturnal episodes, the individual is disoriented with eyes open, and the events may range from ambulating aimlessly, playing a musical instrument, performing inappropriate behavior like urinating in the closet, driving or moving out of the house or resulting in self-injurious action like walking off the balcony. These events can be alarming and disrupt the quality of sleep of their bed partners as well [11].

The perpetual triggers of sleepwalking in the adult population include sleep disorders (restless leg syndrome and sleep apnea), head injury, encephalitis, febrile illness, vitiligo, migraines, stroke and chronic pain syndrome. Besides, the medications that can trigger these events include benzodiazepine receptor agonist (zolpidem-related amnesia), amitriptyline, bupropion, paroxetine, quetiapine, olanzapine, propranolol, metoprolol, topiramate, montelukast and fluoroquinolones [20].

Sleep Terror

Sleep terror or night terror or *pavor nocturnus* is defined as episodes of sudden arousal with increased screaming and crying in fright, along with motor activity and autonomic hyperactivity. The autonomic hyperactivity includes tachypnea, tachycardia, mydriasis and diaphoresis. The patients usually appear terrified and inconsolable and are amnestic or may have a vague recollection of the event. Such episodes can be distressful for the bed partner and family member as well and may disturb their quality of sleep [11].

Sleep-related Eating Disorders

SREDs are defined as recurrent episodes of involuntary binge eating after partial awakening from the sleep. The individual is amnestic of the events and consumes carbohydrate food, chocolates or toxic material like raw meat or pet food. Persistent episodes of SRED may result in weight gain, injuries from mishandling the food, dental caries, diabetes or abnormal lipid levels. While evaluating these patients, it is important to differentiate SREDs from nocturnal eating syndrome and binge eating syndrome. In nocturnal eating syndrome, the individual consciously consumes a large amount of food usually before bedtime, whereas in binge eating syndrome, the individual consumes a high-calorie diet during the entire day followed by unhealthy compensatory mechanisms. Medical conditions that may trigger the SREDs include encephalitis, autoimmune hepatitis, narcolepsy, smoking cessation and substance abuse. Certain drugs that may precipitate SREDs include zolpidem, mirtazapine, quetiapine, lithium and anticholinergic medications [11].

REM Sleep Behavior Disorder

RBDs were first identified as a separate category of parasomnias in 1985 and were termed in 1987. RBDs are characterized by an increased motor activity during the REM sleep as a result of dream enactment behavior. This dream enactment can be in the form of kicking, punching, yelling, jumping or violent behavior correlating with the dream. Such type of behavior may result in self-injury or injury to the bed partner. Most of the time, the individuals remember the dream or have a vague idea of the dream.

RBDs can be associated with various neurologic disorders like pontine stroke, subarachnoid hemorrhage, cerebral neoplasm, α-synucleinopathies, multiple sclerosis and narcolepsy [21-23]. The common medications that can trigger RBD include abrupt withdrawal of sedative-hypnotic medications, selective serotonin reuptake inhibitors (SSRI), tricyclic antidepressants, cholinergic medications, biperiden, monoamine oxidase inhibitor (MAOI) and sudden alcohol withdrawal [24-26]. Some studies have also noted RBD with excessive caffeine and chocolate consumption [27-28]. Although RBDs are more common in adults, recent studies show that RBD can be the first sign in childhood narcolepsy with positive HLA DQB1 *0602 [29].

Nightmares

Nightmares are characterized by complex lucid dream patterns that are frightening for the individual and may result in sudden arousal with a vague recall of the dreams. Frequent nightmares may be induced by stressful or traumatic events or with the use of medications like beta-blockers, levodopa, acetylcholinesterase inhibitors and the sudden discontinuation of REM suppressant medications [30].

Recurrent Isolated Sleep Paralysis

Recurrent isolated sleep paralysis is a benign condition, resulting in the loss of voluntary muscle tone and inability to move on awakening. The possible underlying mechanism for sleep paralysis is the prolongation of REM sleep muscle atonia upon awakening. Sleep paralysis may be precipitated by sleep deprivation or circadian rhythm disturbances like jet lag or shift work disorder [30].

Exploding Head Syndrome

Exploding head syndrome is a benign disorder characterized by sudden loud noise or explosive crashing sound in the head that occurs during the wake-sleep transitions or while awakening in the middle of the night. Such episodes may terrify the individual and are usually accompanied by flashes of light or myoclonic jerks [31]. Patient reassurance is usually the mainstay of management but topiramate, nifedipine and clomipramine may be useful for management [32].

Catathrenia

Catathrenia or sleep-related groaning are recurrent episodes of groaning during sleep without any underlying otolaryngologic or vocal cord abnormalities. These episodes usually occur during the expiration phase in the REM stage of sleep [33]. These complaints are usually brought up by their bed partners as it disrupts their quality of sleep. Recent studies have also reported episodes of catathrenia secondary to sodium oxybate in the management of patients with narcolepsy [34]. Continuous positive airway pressure (CPAP) is found to be effective in the management of catathrenia.

Sexsomnia

Sexsomnia is characterized by unusual sexual behaviors that occur during sleep. These events may range from sexual intercourse, sexual assault, masturbation, sexual vocalizations or fondling the bed partners. Sexsomnia is categorized as NREM parasomnias as they occur during partial arousals in slow-wave sleep (stage three) [35]. Shift work and medications like SSRI may act as a trigger for sexsomnia. These events are also exacerbated in patients with Parkinson’s disease with impulse control disorder. There is limited data on the management of sexsomnia; however, some studies have shown improvement in the symptoms with clonazepam and the use of CPAP for the management of underlying obstructive sleep apnea [11].

Status Dissociatus

Status dissociatus is characterized by complete dissociation between the transition from wakefulness to NREM and REM stages of sleep. During these episodes, most of the patients will have violent behavior ranging from screaming, crying, running, kicking or punching and may last for a few minutes to hours. These episodes are seen commonly in individuals with underlying psychiatric disorders. The possible mechanism for these events is linked to GABA thalamolimbic dysfunction. Status dissociatus can be triggered in alcohol withdrawal, autoimmune encephalitis and α-synucleinopathies. The extreme form of status dissociatus is termed as agrypnia excitata, which is characterized by the loss of NREM architecture including sleep spindles and loss of slow-wave sleep, along with the motor and sympathetic hyperactivity. The behavior marker for agrypnia excitata is *a oneiric stupor*, characterized by semi-purposeful gestures performed in a confused hallucinatory state. These events may be seen in alcohol withdrawal, familial fatal insomnia and Morvan syndrome. The management of the underlying sleep disorders or medical condition may be helpful. Clonazepam, alprazolam, temazepam, carbamazepine and melatonin have shown to be effective in the treatment strategies for dissociative disorders [36].

Diagnosis

A detailed sleep history by the patient, and if possible, by their bed partner is the initial step in the evaluation of parasomnias. Clinicians should also enquire about the underlying medical history, family history, history of substance abuse and current medications used in order to determine the specific trigger for the parasomnias. It is also important to rule out other possible differentials such as nocturnal seizure disorder, psychiatric disorders like post-traumatic stress disorder (PTSD), panic attack and psychogenic spells that can mimic parasomnias. Overnight sleep study or polysomnography (PSG) and video electroencephography (EEG) are useful to identify parasomnias and detect any underlying sleep disorders that may contribute to sleep fragmentation and possible parasomnia [30].

Management

The initial step in the management is to identify and treat any comorbid sleep disorder (obstructive sleep apnea and restless leg syndrome) or medical condition along with the termination of inducing agents (benzodiazepine receptor agonist, antidepressants and antipsychotics) that could be a possible trigger for parasomnias.

Most childhood parasomnias (confusional arousals, sleepwalking, sleep terror and nightmares) are benign and children tend to outgrow them. Hence, in those cases, reassurance and educating the parents can be useful without any medical intervention.

For adult parasomnias, it is very important to educate the patients and their bed partner about the environment safety methods in order to ensure the safety of the individual. Patients should be advised to remove any firearms, sharp objects or furniture near the bed area. Locking the windows and bedroom door alarms can be useful for sleepwalkers. It is very crucial to ensure environment safety techniques in patients with RBDs because of higher chances of injury to self or their bed partners. Patients should be advised to use extra padding or pillows on the sides of the bed, or use padded armrests on the bedsides to prevent falls and injury. The bed partners should be made aware of the risk of injury, and in case of violent behaviors, they should be advised to sleep in a separate bed.

Psychotherapy can be helpful in most NREM parasomnias. Benzodiazepines are the mainstay of management for most persisting parasomnia. Clonazepam is highly effective in the dose of 0.25 to 1 mg in preventing arousals and REM sleep dissociations. For RBDs, clonazepam and melatonin are found to be highly effective. Imipramine, levodopa, carbamazepine and pramipexole have been tried in the past but with limited success in the management [30].

## Conclusions

It is crucial for clinicians to understand the characteristics of various parasomnias and their association with medical and sleep-related disorders. Parasomnias are distressful and can disrupt the lifestyle of the patients and their bed partners. Most of the time, these events are brought up by their bed partners as the patient may be embarrassed or hesitant to discuss them. As clinicians, it is important to reassure the patients and discuss in detail about their sleep history and follow the possible diagnostic approach for a complete evaluation.
